# Supplementary material for: Compositional Effects of Additively Manufactured Refractory High-Entropy Alloys under High-Energy Helium Irradiation
Source: Nanomaterials (Basel). 2022 Jun 10;12(12):2014. doi: 10.3390/nano12122014 (PMC9228246; doi:10.3390/nano12122014)
Supplement: Supplementary file 1 [file nanomaterials-12-02014-s001.zip › nanomaterials-1748291-supplementary.pdf]

Supporting Information

Compositional Effects of Additively Manufactured Refractory High-Entropy Alloys under High-Energy Helium Irradiation

Eric Lang [1,2,*], Kory Burns [1,3], Yongqiang Wang [4], Paul G. Kotula [1], Andrew B. Kustas [1], Sal Rodriguez [1], Assel Aitkaliyeva [3] and Khalid Hattar [1]

[1] Sandia National Laboratories, Albuquerque, NM 87185, USA; kdburns@sandia.gov (K.B.); pgkotul@sandia.gov (P.G.K.); akustas@sandia.gov (A.B.K.); sbrodri@sandia.gov (S.R.); khattar@sandia.gov (K.H.)
[2] Department of Nuclear Engineering, University of New Mexico, Albuquerque, NM 87131, USA
[3] Department of Materials Science and Engineering, University of Florida, Gainesville, FL 32611, USA; aitkaliyeva@mse.ufl.edu
[4] Los Alamos National Laboratory, Los Alamos, NM 87545, USA; yqwang@lanl.gov
* Correspondence: ejlang@sandia.gov

Pre-Irradiation EBSD

Grain sizes and relative orientations were determined via electron backscatter diffraction (EBSD) for the NbTa, MoNbTa, MoNbTaW, and NbTaVW samples. The W sample was unindexable with EBSD and grain size in the He-implanted region was determined via line intercept method on STEM micrographs.

Figure S1 shows EBSD IPF-Z maps of samples, as indicated.

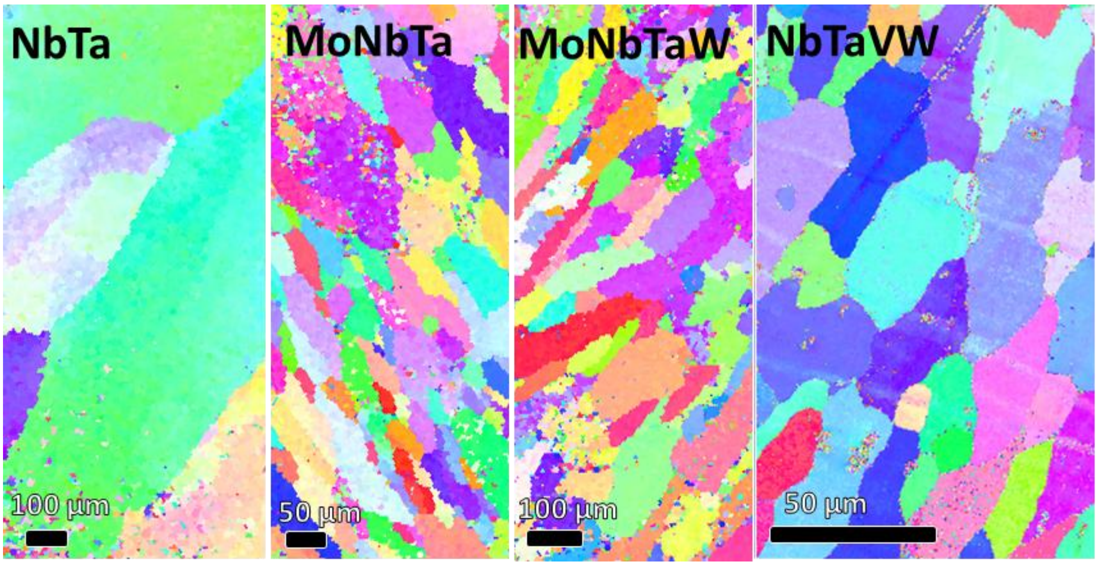

Figure S1. EBSD IPF-Z maps of NbTa, MoNbTa, MoNbTaW, and NbTaVW samples in the as-fabricated states.

Post-Irradiation Microstructures

Following the helium irradiation, SEM analysis showed no new elemental segregation on the samples. Micrographs in Figure S2 show the samples following He implantation, with no blistering evident and no micro-scale segregation of elements observed.

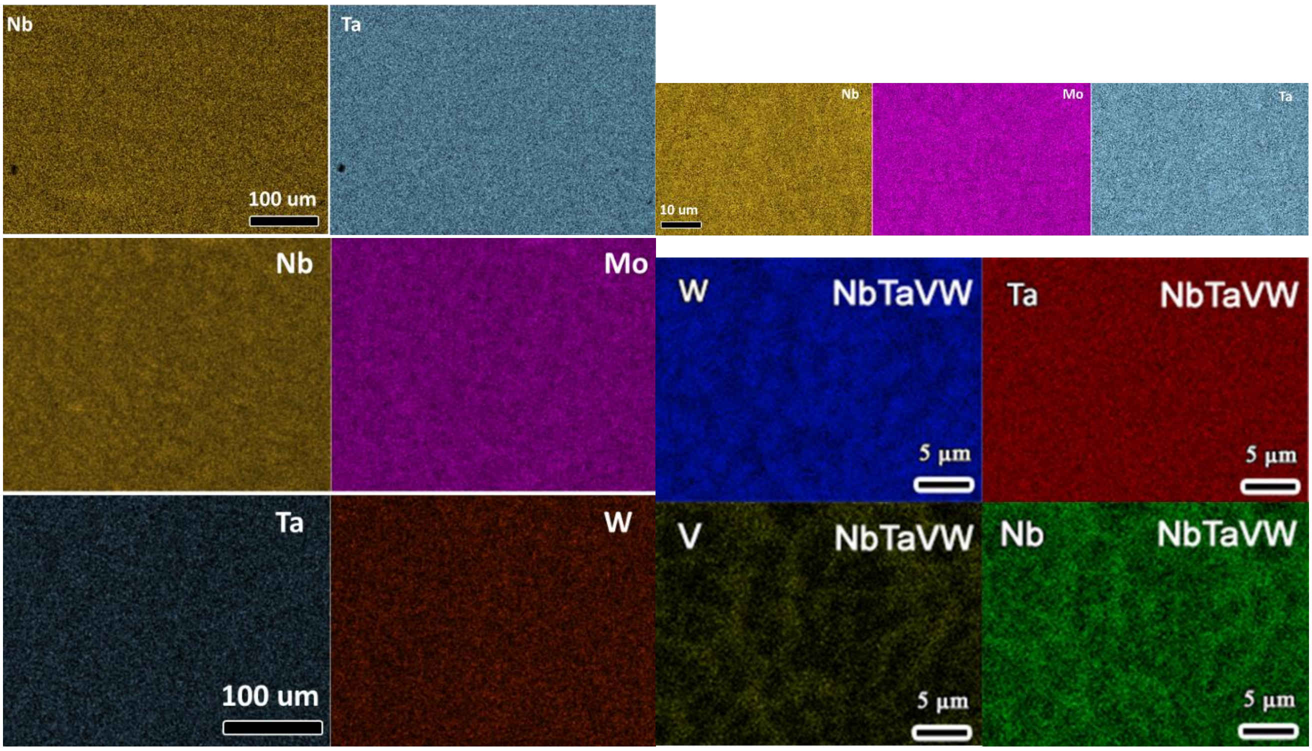

Figure S2. SEM-EDS maps of samples following He implantation showing elemental segregation only in the NbTaVW sample.

Helium Bubble Analysis

The bubble analysis in Python is able to produce profiles of the bubbles, thresholded for depth. In Figure S3 we show the bubbles that were identified by the Python code and used to quantify the size and density of bubbles in the samples. Here, we show that bubbles are identifiable in all samples, despite contrast in the micrographs obscuring the bubbles in the black and white micrographs.

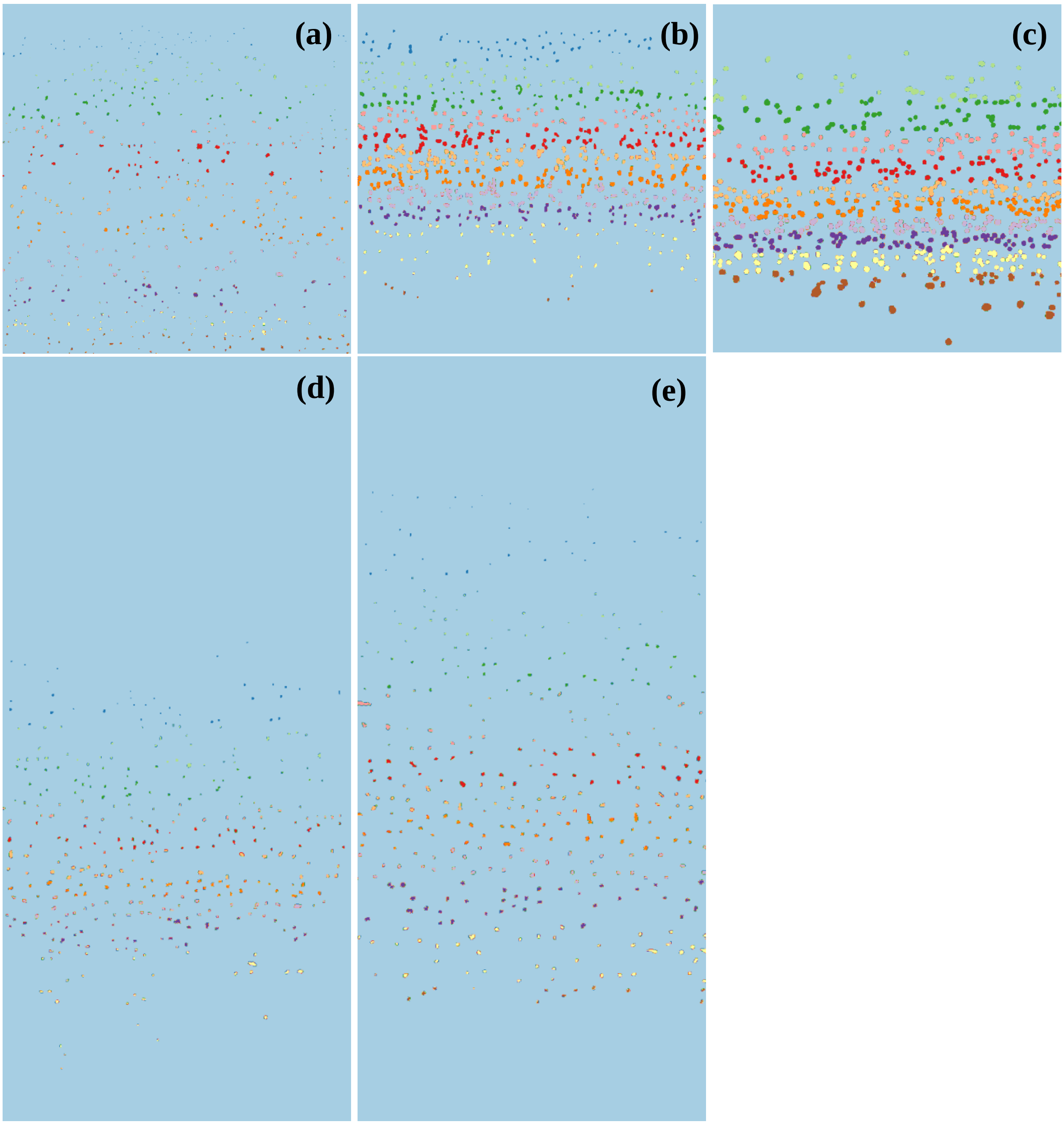

Figure S3. Segmented and thresholded images of He bubbles in STEM micrographs in corresponding samples: (**a**) W, (**b**) NbTa, (**c**) NbTaVW, (**d**) MoNbTa, and (**e**) MoNbTaW.

STEM-EDS was conducted on all implanted alloys to investigate nanoscale elemental segregation following He implantation. Figure S4 shows STEM-EDS maps of all samples following He implantation showing no elemental segregation in the NbTa, MoNbTa, or MoNbTaW samples.

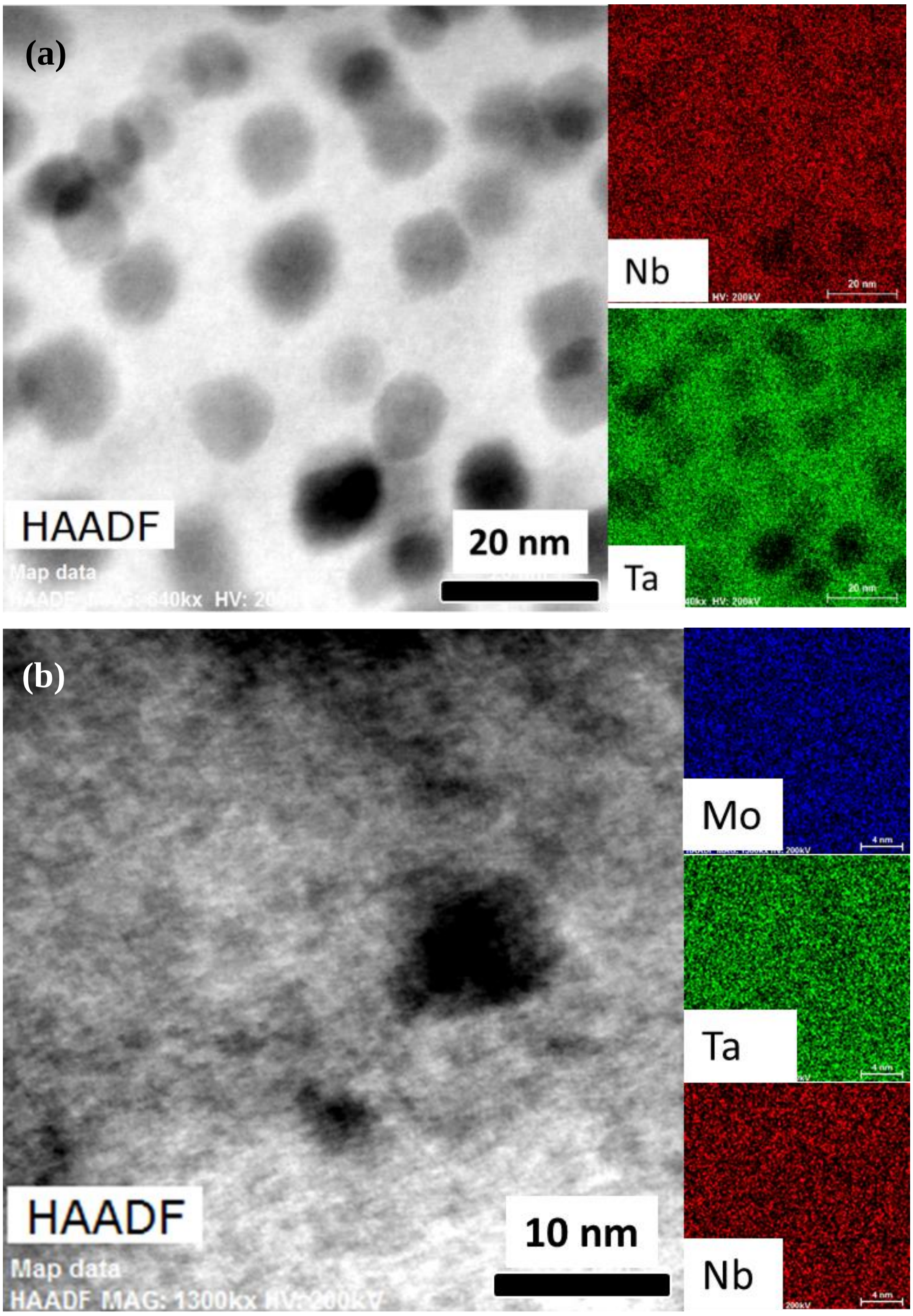

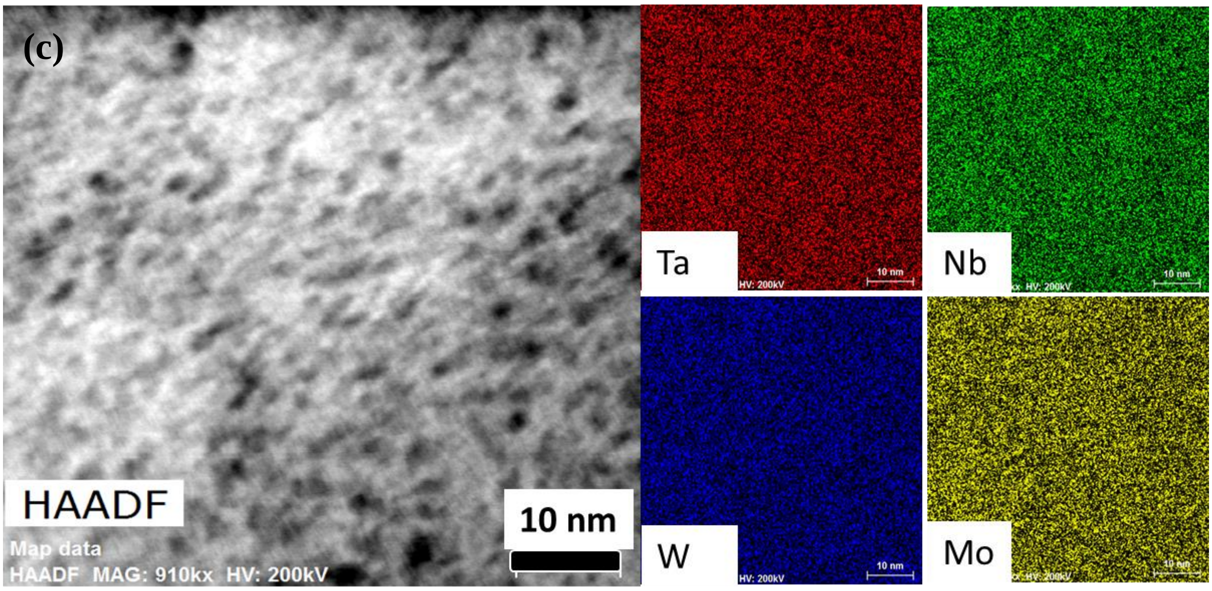

Figure S4: STEM-EDS micrographs of all alloys following He implantation. No elemental segregation is observed in the (a) NbTa, (b) MoNbTa, and (c) MoNbTaW.
